# Comparison of four algorithms on establishing continuous reference intervals for pediatric analytes with age-dependent trend

**DOI:** 10.1186/s12874-020-01021-y

**Published:** 2020-06-01

**Authors:** Kun Li, Lixin Hu, Yaguang Peng, Ruohua Yan, Qiliang Li, Xiaoxia Peng, Wenqi Song, Xin Ni

**Affiliations:** 1 Department of Epidemiology and Health Statistics, School of Public Health, Capital Medical University, Beijing, 100069 China; 2 Center for Clinical Epidemiology and Evidence-based Medicine, Beijing Children’s Hospital, Capital Medical University, National Center for Children Health, No.56 Nanlishi Road, Beijing, 100045 China; 3 Department of Clinical Laboratory Center, Beijing Children’s Hospital, Capital Medical University, National Center for Children Health, No.56 Nanlishi Road, Beijing, 100045 China; 4 Beijing Key Laboratory for Pediatric Diseases of Otolaryngology, Head and Neck, Surgery, Beijing Children’s Hospital, Capital Medical University, National Center for Children Health, No.56 Nanlishi Road, Beijing, 100045 China

**Keywords:** Continuous reference intervals, Pediatric, Graphical report, Clinical laboratory

## Abstract

**Background:**

Continuous reference intervals (RIs) allow for more precise consideration of the dynamic changes of physiological development, which can provide new strategies for the presentation of laboratory test results. Our study aimed to establish continuous RIs using four different simulation methods so that the applicability of different methods could be further understood.

**Methods:**

The data of alkaline phosphatase (ALP) and serum creatinine (Cr) were obtained from the Pediatric Reference Interval in China study (PRINCE), in which healthy children aged 0–19 years were recruited. The improved non-parametric method, the radial smoothing method, the General Additive Model for Location Scale and Shape (GAMLSS), and Lambda-Median-Sigma (LMS) were used to develop continuous RIs. The accuracy and goodness of fit of the continuous RIs were evaluated based on the out of range (OOR) and Akaike Information Criterion (AIC) results.

**Results:**

Samples from 11,517 and 11,544 participants were used to estimate the continuous RIs of ALP and Cr, respectively. Time frames were partitioned to fulfill the following two criteria: sample size = 120 in each subgroup and mean difference = 2 between adjacent time frames. Cubic spline or penalized spline was used for curve smoothing. The RIs estimated by the four methods approximately overlapped. However, more obvious edge effects were shown in the curves fit by the non-parametric methods than the semi-parametric method, which may be attributed to insufficient sample size. The OOR values of all four methods were smaller than 10%.

**Conclusions:**

All four methods could be used to establish continuous RIs. GAMLSS and LMS are more reliable than the other two methods for dealing with edge effects.

## Background

Reference intervals (RIs) are commonly used in medicine to interpret laboratory test results and have been traditionally used in clinical practice to aid diagnosis [1]. Pediatric RIs represent the physiological conditions of normal children and adolescents during development [2]. Dynamic anatomical and physiological development accounts for high variability of many biochemical analytes with increasing age, particularly in the first years of life and during puberty [3]. Therefore, the definitions of pediatric RIs should consider special population features, among which age and sex are the most important for children and adolescents [4].

A familiar method to elucidate trends of age dependence of biochemical analytes is to establish RIs for each age partition. The use of discrete RIs for different age groups is well-established in clinical practice and allows easy integration into current laboratory information systems. To improve the accuracy of age partitioning, an age partitioning algorithm for RI estimation was developed in our previous publication [2]. However, it is still difficult for that model to describe analyte concentrations at the margins of age partitioning, especially abrupt changes during relatively narrow age periods, such as a radical decrease of alkaline phosphatase (ALP) during puberty [5]. Further, it may be difficult to obtain suitable age partitioning points for analytes with continuous upward trends, such as serum creatinine (Cr).

Analogous to other developmental quantities whose relationships with age are routinely analyzed, a continuous description would seem to be more appropriate for laboratory analytes with special age-dependent trends [6]. For example, growth curves were used by the World Health Organization (WHO) to construct child growth standards [7]. The current approaches for establishing continuous RIs can be divided into the non-parametric and semi-parametric method [3, 5, 6, 8, 9]. These different statistical methods of curve simulation could produce different RIs using the same data [1]. Therefore, it is imperative to explore which method is the most appropriate for RI estimation of analytes with various age-dependent trends. To our knowledge, few statistical simulations have been reported to evaluate how well these methods estimate continuous RIs.

Our aim in the present study is to compare the accuracy of continuous RIs established using four different curve simulation methods to better understand these methods’ applicability. The continuous RIs could facilitate the generation of graphical reports in clinical laboratory settings, which could provide quantitative and dynamic assessments of laboratory test results instead of only absolute values [6].

## Methods

### Data source

Data were obtained from the results of the PRINCE study. The eligibility criteria and other detailed information were previously published [10]. In brief, 14,646 healthy children aged 0–19 years were recruited from the northeast (Liaoning Province), north (Beijing Municipality and Hebei Province), northwest (Shaanxi Province), middle (Henan and Hubei provinces), south (Guangdong Province), southwest (Chongqing Municipality and Sichuan Province), and east (Shanghai Municipality and Jiangsu Province) regions of China from January 2017 to August 2018. All participants were confirmed to be eligible based on a questionnaire screening and subsequent physical examination. Considering that the sample size of children aged less than 1 year was limited, we only included healthy children aged 1–19 years. Analyte tests were measured on a Cobas C702 automated biochemistry analyzer (Roche Diagnostics GmbH, Mannherim, Germany) at the Department of Clinical Laboratory Center of Beijing Children’s Hospital, which was the central laboratory of the PRINCE study. Detailed information on quality control was described in the published protocol [10]. The ALP and Cr analytes were selected from 13 eligible biochemical markers as typical cases because of their special age-dependent trends in children and adolescents. The study was exempted by the Ethics Committee of Beijing Children’s Hospital, affiliated with Capital Medical University, Beijing, China.

### Data cleaning and management

Data cleaning was performed to detect missing values and outliers. Missing values were defined as incomplete information of age, sex, or biochemical analytes. Considering that ALP and Cr are known to vary significantly by age and sex [6], outliers are removed according to sex and age groups (for each 1-year) by Tukey’s method [4]. In this method, outliers are removed if they are less than Q_1_–1.5 × IQR or more than Q_3_ + 1.5 × IQR, in which Q_1_ and Q_3_ are the 25th and 75th percentiles, respectively. IQR is interquartile range, calculated by Q_3_ − Q_1_, where the data have a Gaussian distribution. Otherwise, the data should be transformed by the Box-cox method, expressed by the following formula:

1 $$\mathrm{y}=\left\{\begin{array}{cc}\left({\mathrm{x}}^{\uplambda}-1\right)/\uplambda & for\ \lambda \ne 0\\ {}\ln \left(\mathrm{x}+\mathrm{c}\right)& \mathrm{f} or\ \lambda =0\end{array}\right.$$

where *x* is the original value, *y* is the value after Box-cox transformation, and λ and *c* are parameters calculated by maximum likelihood estimation.

### Statistical simulations

All statistical analysis was performed using SAS 9.4 and R 3.5.1. The lower limit and upper limit values of RIs were calculated as the 2.5 and 97.5% quantiles of the corresponding populations, respectively. Four methods were implemented in this study: the improved non-parametric method, the radial smoothing method (RS), the General Additive Model for Location Scale and Shape method (GAMLSS), and the Lambda-Median-Sigma method (LMS) [4, 8, 11–13]. Both the improved non-parametric method and RS are considered as non-parametric methods because non-parametric curve estimation methods are used during the RI establishment procedure. Although GAMLSS and LMS use smoothing methods in model terms, they are deemed semi-parametric methods because the response variable requires the assumption of a parametric distribution.

In the past decades, several studies have used spline or piecewise polynomial methods to establish continuous RIs of laboratory analytes for interpretation of the age dynamics of children’s development [5, 6, 14]. These studies’ methods can be divided into three steps. First, the whole dataset was split into several age groups; then, discrete RIs were calculated for each age group; finally, the RIs’ limit values for each age group were fit using appropriate smoothing methods, such as spline or polynomial methods [4, 6]. Arzideh et al. optimized the age group classification procedure [3, 15]. They split the whole dataset into overlapping time frames, which allows more precise consideration of rapid changes in analyte concentrations with increasing age. We call Arzideh’s method the improved non-parametric method in the present study and used the bootstrap method to calculate the reference limits for each time frame [3, 16]. To find the most suitable smoothing method for the improved non-parametric method, cubic spline, penalized spline, and fractional polynomial smoothing were performed, and the goodness of fit was evaluated by Akaike information criterion (AIC) values calculated under each model [17]. The formula for AIC is as follows.

2 $$AIC=-2 lon\hat{\theta}+k$$

where $\hat{\theta}$ is the maximized log likelihood function, and *k* is the number of effective degrees of freedom used in the model, e.g., *k* = 2. The model with the smallest AIC value is considered to have the best fit.

The LMS model contains three parameters: skewness (L) accounts for deviation from the normal distribution after Box-Cox transformation; the median (M) models the outcome variable depending on one explanatory variable; and the coefficient of variation (S) accounts for variation of data points around the mean and adjusts for non-uniform dispersion [9]. GAMLSS is an extension of the LMS method, which was introduced by Rigby and Stasinopoulos as a way of overcoming some of the limitations associated with generalized linear models and generalized additive models [11, 18]. In contrast with LMS, GAMLSS can accommodate more than one covariate and distribution [11, 19]. The Box-Cox t and Box-Cox power exponential distributions were compared to select the most appropriate type of GAMLSS model [20, 21]. Worm plots were used to assess the fitting results of additive terms and to judge whether simulation of kurtosis was required [22]. The procedure was implemented in the GAMLSS package (version 5.1–2) of the R statistical software package.

In the RS method, various spline functions, such as B-spline and truncated polynomial functions, can be used as the basis function to fit non-parametric curve estimation [8]. Radial bases are sometimes preferred for higher-dimensional problems because of their straightforward extension. Wan XH et al. provided a percentile curve for calculation of arithmetic based on four moments and the Edgeworth-Cornish-Fisher expansion, which was used for some of the present study’s simulations [23].

Before statistical simulation, the whole dataset was randomly partitioned into training and test datasets in an 8:2 ratio. The training dataset were used for model fitting and model selection, and the test dataset were used for assessment of the model’s predictive power to fit training data, i.e., the out of range (OOR) percentage. Considering sex differences in analyte concentrations, the data were divided by sex before the training and test datasets were generated. The process of data set partitioning and RI calculation was repeated 100 times for both ALP and Cr to reduce the random error caused by running too few statistical simulations. In addition, the Wilcoxon test was used to compare whether the training and test datasets had similar age distributions. When *P* ≥ 0.05, the results of dataset partitioning were deemed valid, and otherwise, the partitioning procedure was repeated. According to the recommendations of the Clinical and Laboratory Standards Institute, RIs may be considered valid when the OOR value is < 10% [4], considering that we estimated RIs using 95% intervals. Thus, OOR values close to 2.5% for both the upper and lower reference limits were appropriate for method selection. Furthermore, AIC values were calculated to evaluate the different models’ goodness of fit under the GAMLSS method. Then, the accuracy and goodness of fit of continuous RIs were compared comprehensively based on the OOR and AIC values. The statistical simulation process is summarized in Supplemental Figure 1.

## Results

### Characteristics of ALP and Cr distributions

**Fig. 1:**
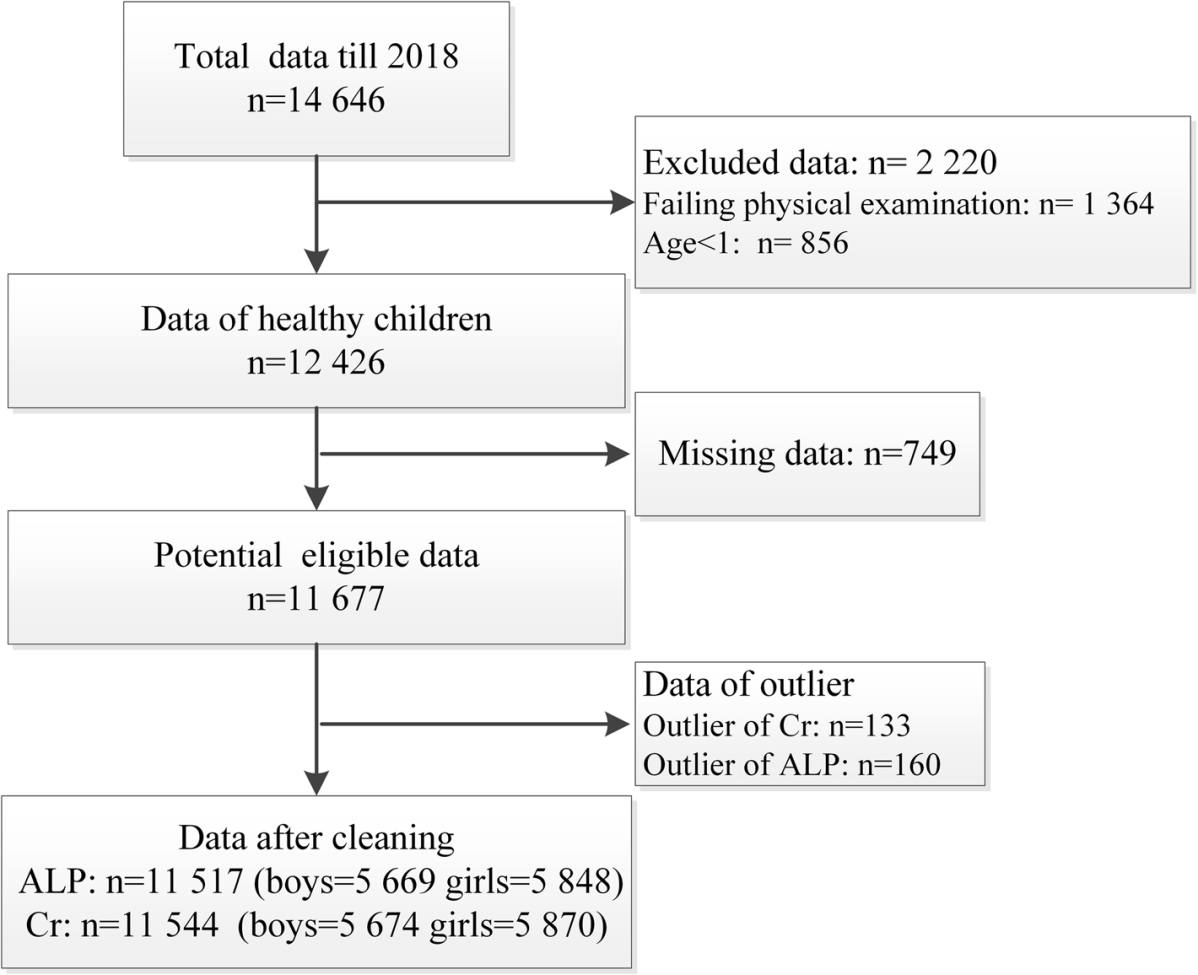
Data cleaning procedure. ALP, alkaline phosphatase; Cr, serum creatinine

The entire data cleaning process is shown in Fig. 1. Scatter diagrams show outliers that were removed by Tukey’s method (Supplemental Figure 2). After data cleaning, samples from 11,517 and 11,544 participants aged 1–19 years were included to calculate the RIs of ALP and Cr, respectively. The distributions of ALP and Cr by age and sex are shown in Table 1. The probability density plots had the same age distributions between the training and test datasets (Supplemental Figure 3).

**Table 1:** The distributions of alkaline phosphatase and serum creatinine by age and sex

Age (years)	Alkaline phosphatase (U/L)						Serum creatinine (umol/L)					
	Boys			Girls			Boys			Girls		
	***n***	Mean	SD	***n***	Mean	SD	***n***	Mean	SD	***n***	Mean	SD
**> = 1**	255	253.96	62.55	225	243.68	50.98	260	24.85	3.52	227	23.78	3.33
**2~**	304	228.59	47.73	266	236.15	55.18	309	28.58	4.02	273	28.61	4.46
**3~**	446	222.06	45.83	337	220.36	45.17	449	32.61	4.23	328	31.08	3.50
**4~**	287	214.88	42.28	298	224.49	47.75	286	35.14	4.36	300	34.24	4.34
**5~**	286	226.13	47.68	280	233.63	47.95	289	37.18	4.32	284	36.95	4.50
**6~**	393	227.92	43.78	355	230.68	47.52	392	40.90	5.34	362	40.02	4.99
**7~**	413	231.92	52.08	372	237.97	51.65	409	41.93	4.95	378	40.93	5.02
**8~**	401	240.51	53.58	405	250.31	58.60	402	44.04	5.41	405	43.02	5.59
**9~**	396	247.88	58.34	371	261.05	64.72	386	46.33	5.14	369	45.15	5.23
**10~**	426	256.03	61.68	390	283.31	61.18	422	48.99	5.85	391	45.36	5.19
**11~**	298	283.69	75.29	287	272.87	68.94	299	49.87	6.12	293	45.75	6.25
**12~**	408	325.02	92.31	356	226.99	83.11	410	55.62	8.52	353	51.64	7.93
**13~**	283	319.35	97.79	315	178.50	68.13	282	59.66	9.65	319	53.46	8.10
**14~**	230	266.79	99.06	239	137.99	49.86	232	66.92	11.03	240	55.65	7.73
**15~**	309	178.57	80.91	383	100.32	33.80	307	75.27	10.84	375	60.77	7.39
**16~**	196	132.40	49.03	244	87.52	24.61	198	77.84	11.09	247	61.43	8.25
**17~**	162	103.91	28.26	264	74.55	18.27	164	79.58	10.25	264	61.55	7.87
**18~**	113	91.61	18.18	293	70.80	15.47	115	78.51	9.76	297	60.16	7.92
**19–20**	63	83.44	19.79	168	69.28	14.40	63	84.51	8.72	165	59.67	6.85

**Fig. 2:**
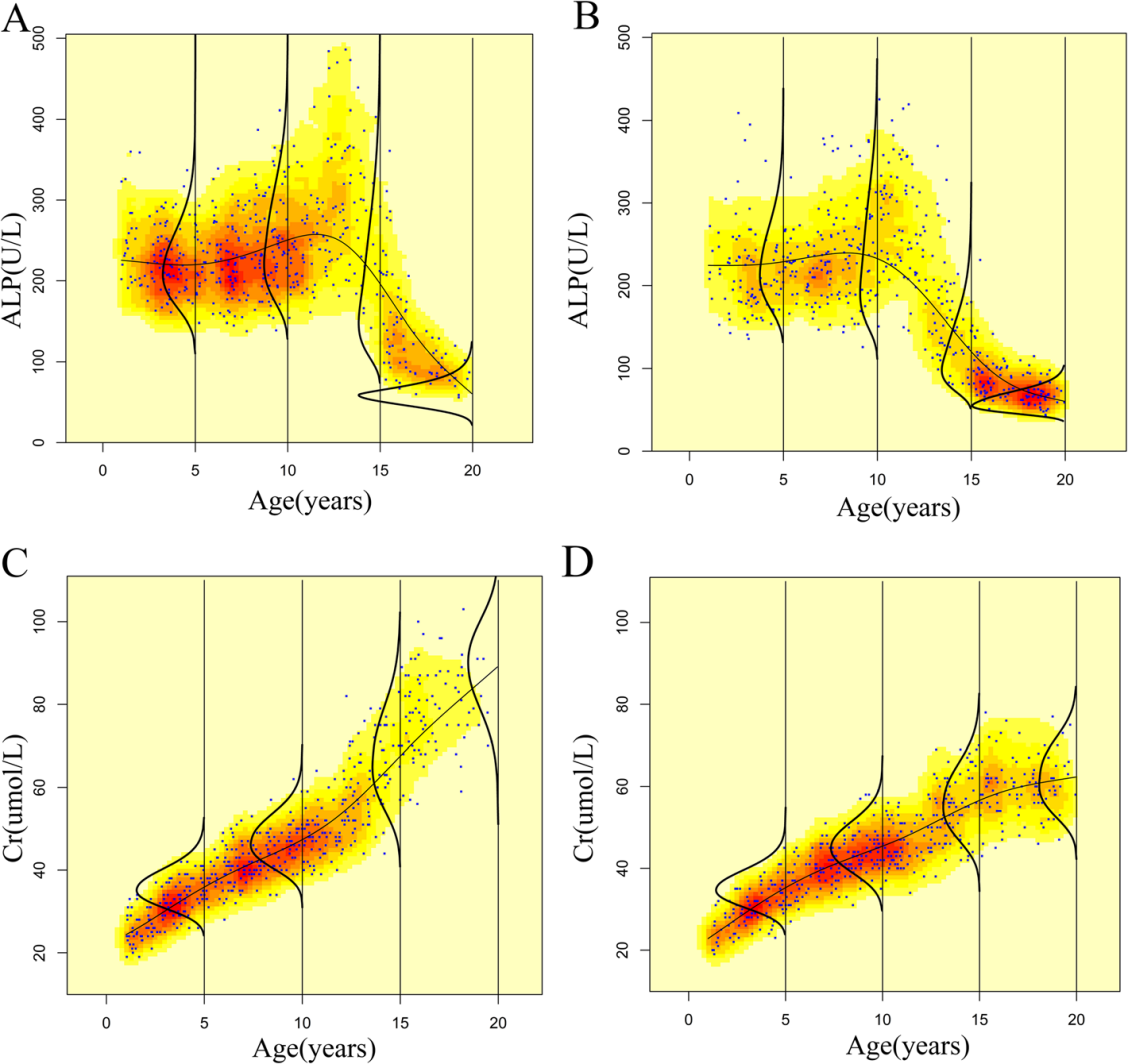
The age dependent trend of alkaline phosphatase and serum creatinine by sex. **a**. alkaline phosphatase of boys. **b**. alkaline phosphatase of girls. **c**. serum creatinine of boys. **d**. serum creatinine of girls. ALP, alkaline phosphatase; Cr, serum creatinine. Notes: The center lines are fitted by GAMLSS method, the other curves are probability density functions and the horizontal axis represents the probability density for each age group. The density of the data points is represented by the color chromaticity

We represented the density of the data points by color chromaticity using the *plotSimpleGamlss* function in R. The results demonstrated that girls were more concentrated in the adolescent groups than boys (Fig. 2). Additionally, significant age dependence was shown in the trends of ALP and Cr, and the results differed between the two analytes. For example, Cr continuously increased with age from 1 to 19 years, whereas a sharp decrease in ALP was observed after puberty (age 12 and 14 years for girls and boys, respectively). Cr showed the same tendency between boys and girls throughout the childhood phase, where boys plateau later than girls after a long period of growth. However, boys’ and girls’ ALP levels showed a decreasing trend in the first 4 years of life, but the levels then increased until puberty.

### Simulation of time frames for the improved non-parametric method

**Fig. 3:**
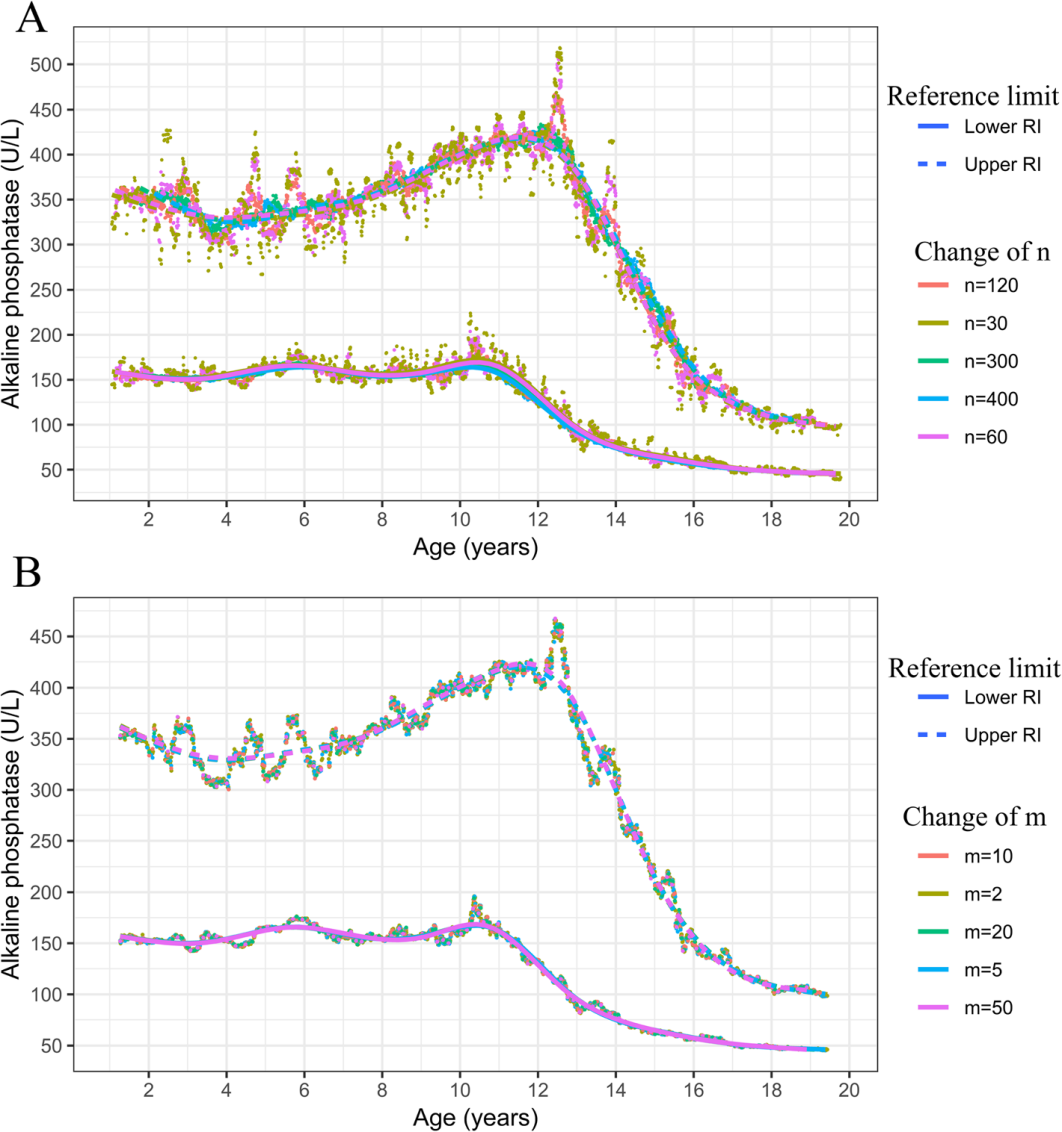
Simulation of time frames with different sample sizes (n) and mean difference between adjacent time frames (m). **a.** Simulation of time frames with different sample sizes (n), n = sample size in time frame. **b**. Simulation of mean difference between adjacent time frames, m = mean difference between adjacent time frames. ALP, alkaline phosphatase. Notes: Lines are fitted by cubic splines

The balance between the sample size in each time frame and the number of time frames was difficult to maintain. Through a process of statistical simulation, we obtained a figure with changing values of n and m (n = sample size in time frame, m = mean difference between adjacent time frames), shown in Fig. 3. Although the curves obtained under various parameters were similar, we found that when the sample size is small (e.g., *n* < 60), there were more discrete reference limit values, which may drift, influencing the curve fitting results. Moreover, when the sample size was too large (e.g. *n* > 300), some details of the curve were lost, especially for ALP at ages 14–16 years, which showed a cliff-like descent. The Clinical and Laboratory Standards Institute (CLSI) recommends a minimum sample size of 120 to establish RIs. Combining CLSI’s suggestion with Pavlov’s research [16], we finally set the sample size within each time frame to 120. Using an excessive number of time frames increases the arithmetic load. Therefore, the n and m parameters were set as 120 and 2, respectively, for both ALP and Cr.

### Simulation of smoothing methods for the improved non-parametric method

The AIC values for three smoothing methods are shown in Supplemental Table 1. Smoothing parameters were selected by internal (i.e., local) maximum likelihood estimation in the R software package [24]. Among all models, the penalized spline method had the smallest AIC value. The continuous RIs fitted by penalized spline, cubic spline, and fraction polynomials are shown in Supplemental Figure 4. The fraction polynomials did not fit well at the end of the curve for ALP, and there was a cross between the upper and lower percentile curves. Furthermore, fluctuation occurred in the smooth curve simulated by the penalized spline method. Therefore, we adjusted the smoothing parameters of the cubic spline and penalized spline methods through visual inspection.

### Simulations based on GAMLSS and LMS

Using the GAMLSS method, the four models were simulated using two distribution types (Box-Cox t and Box-Cox power exponential) and two smoothing methods (cubic spline or penalized spline). GAMLSS models’ AIC values are shown in Supplemental Table 2. Compared with the cubic spline smoothing technique, penalized spline fit the data better according to the AIC value, similar to the simulation results of the non-parametric methods. The worm plots of simulations based on the LMS and GAMLSS methods demonstrate that the GAMLSS models fit the data better than LMS, especially for ALP (Supplemental Figure 5). This is because the GAMLSS model is more consistent with the theoretical distribution, in which data points are uniformly distributed on both sides of the center line [22].

### Continuous RIs for pediatric ALP and Cr

**Fig. 4:**
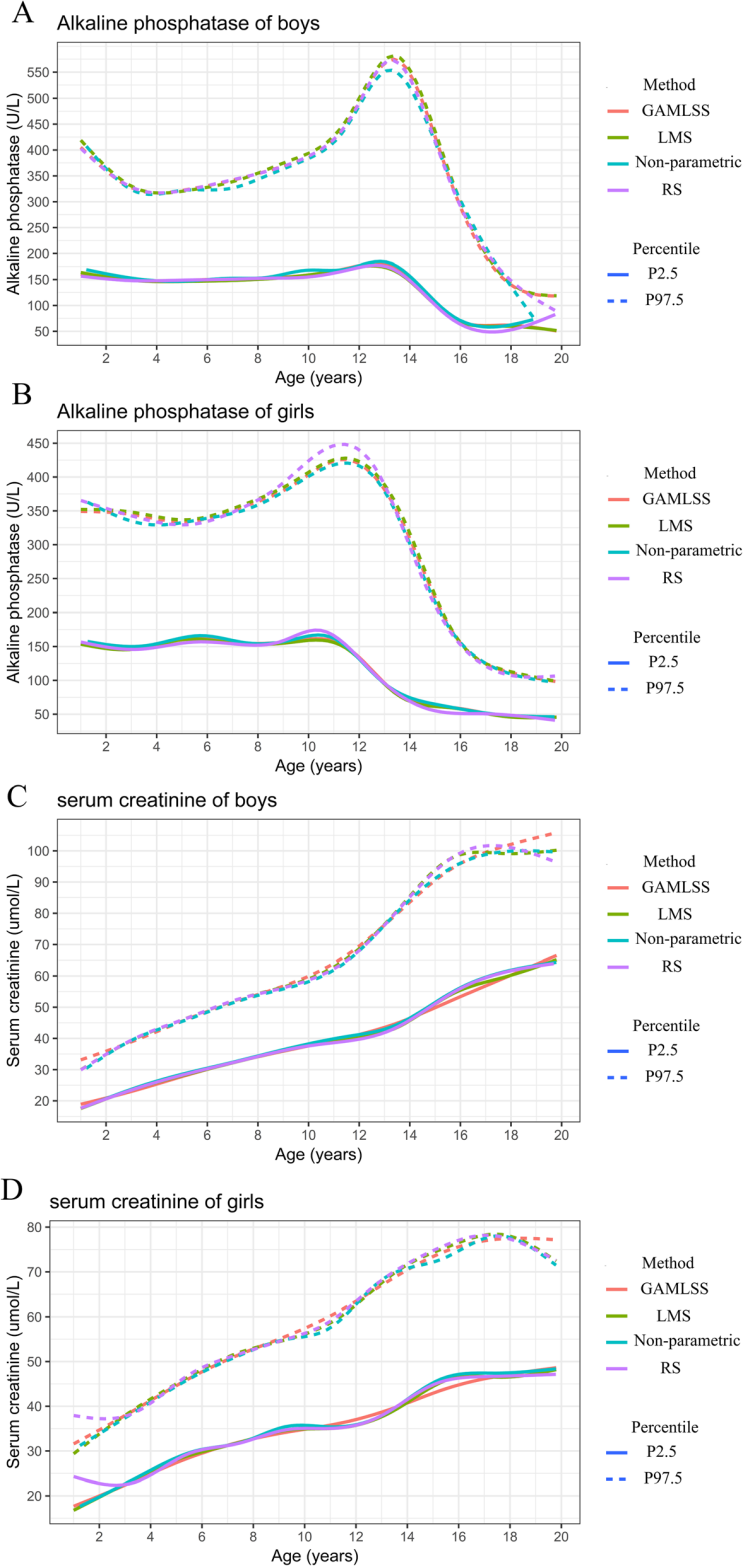
Continuous RIs of serum creatinine and alkaline phosphatase by sex. **a**. alkaline phosphatase of boys. **b**. alkaline phosphatase of girls. **c**. serum creatinine of boys. **d**. serum creatinine of girls. GAMLSS, General Additive Model for Location Scale and Shape method; LMS, Lambda-Median-Sigma method; RS, radial smoothing method; ALP, alkaline phosphatase; Cr, serum creatinine

The continuous RIs were estimated using the improved non-parametric method, the RS method, the GAMLSS method, and the LMS method. Figure 4 shows the results of continuous RI estimation for Cr and ALP using the four methods. The RIs estimated by the LMS, GAMLSS, and RS methods approximately overlapped, while the improved non-parametric method seemed better after visual inspection of the smoothing parameters. However, there were slight differences at the ends and peaks of the curves. Large edge effects were found in the curves fit by the RS method: left and right edge effects appeared for Cr and ALP, respectively. Age-specific reference values found for ALP and Cr using the GAMLSS method are presented in Tables 2 and 3.

**Table 2:** Age-specific reference values for alkaline phosphatase

	Percentiles													
	Alkaline phosphatase of boys (U/L)							Alkaline phosphatase of girls (U/L)						
Age (years)	2.5th	10th	25th	50th	75th	90th	97.5th	2.5th	10th	25th	50th	75th	90th	97.5th
**> = 1**	158.12^a^	183.98	211.87	249.58	295.47	344.57	410.22	147.65	170.83	194.89	226.17	260.05	290.23	323.58
**2~**	154.04	176.04	199.47	230.77	267.96	306.45	356.10	150.59	172.39	196.48	230.20	270.07	308.80	355.40
**3~**	149.78	169.74	190.93	219.21	252.45	286.19	328.80	149.71	169.01	190.69	221.77	259.78	298.16	346.36
**4~**	145.69	164.91	185.44	213.02	245.43	277.99	318.70	149.40	166.49	185.77	213.65	248.26	283.90	329.80
**5~**	146.41	165.46	185.90	213.56	246.01	278.29	318.22	159.86	176.36	195.03	222.15	256.15	291.68	338.35
**6~**	154.68	174.62	196.14	225.44	259.80	293.67	335.16	165.39	182.25	201.18	228.42	262.13	296.88	341.79
**7~**	149.28	169.46	191.51	221.98	258.00	293.50	336.94	156.96	175.67	196.59	226.41	262.79	299.55	345.84
**8~**	151.66	173.21	197.06	230.53	270.47	309.86	358.01	152.36	174.93	199.89	234.82	276.26	316.78	365.84
**9~**	156.19	178.24	202.77	237.41	278.74	319.15	368.11	153.30	180.38	209.44	248.57	292.80	333.93	381.25
**10~**	159.57	182.49	208.21	244.90	288.83	331.60	383.14	163.50	194.82	227.33	269.50	315.23	356.14	401.53
**11~**	163.55	188.80	217.60	259.44	310.24	359.94	420.05	163.59	197.41	232.63	278.44	328.23	372.88	422.47
**12~**	177.13	206.76	241.16	292.20	355.15	417.23	492.69	128.62	160.81	196.43	245.75	303.01	357.46	421.22
**13~**	183.35	217.16	257.27	318.29	395.16	471.87	566.02	93.35	117.65	146.58	190.35	246.77	306.19	383.20
**14~**	149.89	181.66	220.51	281.75	361.49	442.93	544.83	74.21	90.45	110.36	141.94	185.71	236.02	308.37
**15~**	100.65	124.89	155.45	205.40	272.68	343.05	432.82	63.30	74.12	87.20	107.79	136.42	169.87	219.54
**16~**	71.07	87.33	107.74	140.90	184.96	230.14	286.59	55.64	63.43	72.59	86.56	105.25	126.27	156.26
**17~**	62.80	73.95	87.40	108.24	134.33	159.48	189.13	50.72	57.23	64.69	75.68	89.73	104.74	124.89
**18~**	59.95	68.26	77.98	92.52	109.90	125.85	143.82	47.53	53.67	60.54	70.35	82.37	94.60	110.11
**19~**	56.06	62.90	70.82	82.53	96.23	108.52	122.04	45.38	51.64	58.49	67.96	79.08	89.88	102.90

^a^ Calculated by GAMLSS method

**Table 3:** Age-specific reference values for creatinine

	Percentiles													
	Serum creatinine of boys (umol/L)							Serum creatinine of girls (umol/L)						
Age (years)	2.5th	10th	25th	50th	75th	90th	97.5th	2.5th	10th	25th	50th	75th	90th	97.5th
**> = 1**	19.01^a^	21.11	23.18	25.72	28.37	30.71	33.28	17.75	19.83	21.78	24.03	26.43	28.79	31.65
**2~**	20.84	22.97	25.09	27.71	30.49	33.02	35.85	19.96	22.13	24.18	26.57	29.13	31.63	34.67
**3~**	23.01	25.20	27.38	30.09	33.03	35.76	38.92	22.42	24.66	26.81	29.35	32.09	34.74	37.93
**4~**	25.38	27.64	29.90	32.72	35.81	38.75	42.22	24.94	27.26	29.52	32.23	35.16	37.96	41.28
**5~**	27.77	30.13	32.49	35.43	38.67	41.78	45.48	27.36	29.78	32.15	35.05	38.18	41.13	44.60
**6~**	30.07	32.54	35.02	38.10	41.48	44.72	48.59	29.51	32.07	34.58	37.66	40.97	44.08	47.70
**7~**	32.20	34.80	37.38	40.60	44.13	47.48	51.44	31.29	34.02	36.69	39.94	43.42	46.66	50.42
**8~**	34.14	36.84	39.54	42.91	46.59	50.04	54.06	32.72	35.64	38.48	41.91	45.55	48.92	52.80
**9~**	35.91	38.72	41.56	45.14	49.03	52.62	56.73	33.89	37.03	40.06	43.70	47.53	51.04	55.04
**10~**	37.60	40.55	43.58	47.50	51.75	55.59	59.89	34.85	38.27	41.55	45.47	49.55	53.24	57.39
**11~**	39.30	42.47	45.81	50.23	55.07	59.33	64.01	35.78	39.53	43.12	47.41	51.83	55.78	60.15
**12~**	41.24	44.75	48.52	53.63	59.25	64.16	69.50	37.00	41.08	45.00	49.68	54.50	58.77	63.47
**13~**	43.58	47.59	51.92	57.83	64.35	70.07	76.27	38.69	43.00	47.16	52.20	57.41	62.00	67.04
**14~**	46.41	51.07	56.04	62.73	70.06	76.52	83.56	40.74	45.14	49.45	54.73	60.24	65.10	70.43
**15~**	49.77	55.12	60.68	67.96	75.84	82.79	90.42	42.89	47.27	51.63	57.06	62.77	67.82	73.38
**16~**	53.41	59.41	65.43	72.98	81.00	88.11	95.95	44.78	49.09	53.43	58.90	64.72	69.90	75.64
**17~**	56.89	63.51	69.84	77.38	85.14	92.02	99.62	46.17	50.41	54.69	60.11	65.91	71.14	76.98
**18~**	60.21	67.38	73.88	81.22	88.53	94.97	102.02	47.15	51.31	55.50	60.79	66.48	71.64	77.46
**19–20**	63.66	71.26	77.81	84.86	91.68	97.65	104.14	47.99	52.01	56.07	61.19	66.72	71.73	77.40

^a^ Calculated by GAMLSS method

**Fig. 5:**
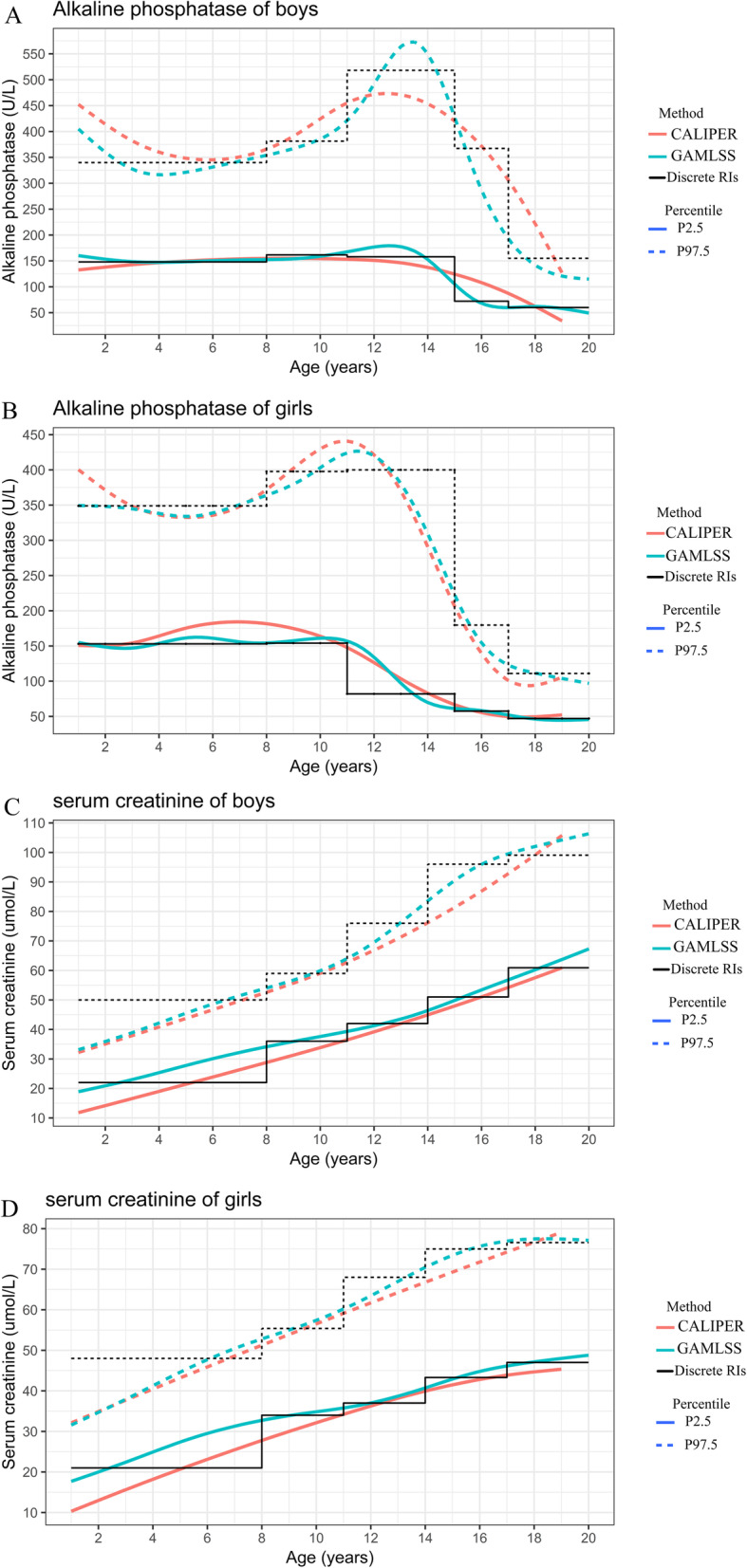
Comparing the RIs with CALIPER study**. a**. alkaline phosphatase of boys. **b**. alkaline phosphatase of girls. **c**. serum creatinine of boys. **d**. serum creatinine of girls. ALP, alkaline phosphatase; Cr, serum creatinine; GAMLSS, General Additive Model for Location Scale and Shape method; CALIPER: Canadian Laboratory Initiative in Pediatric Reference Intervals

Figure 5 shows the differences between discrete RIs partitioned by the decision tree technique and continuous RIs calculated by the GAMLSS method. The discrete RIs presented a ladder shape that jumped several times with increasing age. In addition, we added the continuous RIs from the CALIPER study [25]. The curves from CALIPER were smoother than this study’s GAMLSS method results, especially for ALP in boys. Further, both the upper and lower reference limits of Cr calculated by CALIPER were slightly lower than those in the present study.

### Verifying RIs by test set

The continuous RIs were verified using the test dataset. All OOR values in Table 4 were smaller than 10%. The OOR percentages of the LMS, GAMLSS, and RS methods were much closer to 5%, and both the lower OOR and upper OOR proportions were both close to 2.5%. We also verified the continuous RIs calculated by CALIPER, all OOR values were less than 10%, except for ALP of girls. In addition, we calculated the OOR rates of discrete RIs, which were also close to 5%. Moreover, we created a table of OOR values for each year of age to ensure that the RIs accurately represent the relationship between age and analyte concentration (Supplemental Tables 3 and 4), which clearly showed the differences between continuous and discrete RIs. Although this study’s sample size may be relatively insufficient, the OOR values of discrete RIs have larger variation in each age group compared with those of continuous RIs, especially near the thresholds of age divisions.

**Table 4:** Out of range (OOR) of different simulation methods verified with test data set

Model	Total of OOR (%)	Lower OOR (%)	Upper OOR (%)
**Serum creatinine of boys**			
Improved non-parametric^a^	6.50^b^	3.34	3.16
RS	4.87	2.39	2.47
LMS	5.18	2.62	2.57
GAMLSS^c^	5.03	2.38	2.65
Discrete reference interval	4.32	2.08	2.25
CALIPER	7.58	0.57	7.01
**Serum creatinine of girls**			
Improved Non-parametric	6.62	3.34	3.28
RS	5.21	2.60	2.62
LMS	4.94	2.48	2.46
GAMLSS	5.45	2.85	2.60
Discrete reference interval	4.17	2.00	2.18
CALIPER	6.13	0.84	5.28
**Alkaline phosphatase of boys**			
Improved non-parametric	7.01	3.69	3.31
RS	5.26	2.75	2.50
LMS	4.74	2.46	2.29
GAMLSS	5.04	2.51	2.53
Discrete reference interval	5.66	3.03	2.63
CALIPER	6.81	4.41	2.39
**Alkaline phosphatase of girls**			
Improved non-parametric	6.72	3.59	3.13
RS	4.53	2.30	2.23
LMS	4.94	2.48	2.46
GAMLSS	5.02	2.43	2.59
Discrete reference interval	4.90	2.32	2.59
CALIPER	10.22	7.10	3.12

*OOR* Out of range, *GAMLSS* General Additive Model for Location Scale and Shape method, *CALIPER* Canadian Laboratory Initiative in Pediatric Reference Intervals, *LMS* Lambda-Median-Sigma method, *RS* radial smoothing method

^a^ Smoothing parameters are selected by visual inspection of non-parametric method

^b^ All values are calculated as mean of the results of 100 times simulation

^c^ The models of GAMLSS method are selected from Table 3, which AIC is smallest

## Discussion

Age partitioning is a common issue not only for pediatric RIs but also for other clinical laboratory indexes [2, 3, 26, 27]. However, the use of age portioning methods to establish RIs still has some limitations, as the use of discrete age groups does not sensitively reflect continuous changes in growth and development. This problem is illustrated in Fig. 5. In contrast to the discrete RIs, the continuous RIs allow a precise representation of age and sex-dependent change during growth and development. Therefore, to provide evidence for the applicability of different algorithms to establish continuous RIs, we presented continuous RIs simulated by four methods from infancy to adulthood.

The age-dependent trends of ALP and Cr in the present study were consistent with those in previous studies, which represent distinctive age-dependent trends [6, 25]. Different from study reported by Zierk [6], in which data were collected from hospitals, all the reference individuals were healthy children recruited for the PRINCE study. Moreover, there were slight differences in the continuous RIs between the CALIPER study and the present study: the reference limits were slightly higher in the present study, which may be caused by differences in the reference population and inspection instruments.

Establishing a reference interval using a non-parametric method is an indirect process: curve fitting is simulated considering only the values of discrete reference limits, rather than including all data. This weakness leads to curve fluctuations, even if it has the best AIC value and appropriate smoothing parameters. Our research indicates that although the model can obtain a better AIC value, the smoothing parameters adjusted by visual inspection better represent the whole dataset and are more suitable in the non-parametric methods. Therefore, the trends of age dependence should be fully understood before establishing continuous RIs using the non-parametric methods. Moreover, it is necessary to adjust the smoothing parameters through visual inspection instead of only relying on software algorithms.

We used a robust bootstrap method to estimate the discrete RIs for each time frame in the improved non-parametric method, after considering the accuracy and feasibility of various methods. In our study, not all analyte levels had normal distributions across the 200,000 time frames. Therefore, the data should be transformed to a normal distribution if we use parametric methods to calculate RIs. However, hypotheses testing and data transformation for large datasets depend on programming capabilities and statistical package functions. We tried to use the *powertransform* function in R to perform the Box-Cox transformation, but there were still some time frames for which the best λ could not be obtained, and those needed to be debugged manually. However, manual debugging would incur an inestimable time cost. According Pavlov’s research [16], the bootstrap method has relatively high accuracy when the sample size is relatively small, so we chose the bootstrap method for the improved non-parametric procedure.

Additionally, LMS and GAMLSS have been widely applied to establish growth curves. They also perform well at establishing continuous RIs for analytes. As presented, the OOR percentages of those two methods were close to 5%. In contrast, the OOR proportions of the non-parametric methods were more than 6% for ALP, which means that the non-parametric methods’ RIs are narrower than those of the LMS and GAMLSS methods. Further, both the GAMLSS and LMS methods are simple to implement and adapt to complex age-dependent trends, especially when the age distribution of the analyte’s concentration is not fully understood.

As a new approach for estimation of age-specific reference percentile curves, the RS method performs well at growth curve establishment [8]. However, it did not generate effective RIs for ALP without data transformation by the Box-Cox method (Supplemental Figure 6). This means that the distribution of data should be approximately normal, especially when the analyte has a more intricate age-dependent trend.

As for verification results, all four methods’ OOR values were less than 10%, which means that all methods showed good fit for establishing continuous RIs of Cr and ALP. However, edge effects were observed in all of the curves fit by these four methods. Even if the smoothing parameters were adjusted by visual inspection, the drift at the end of the curve was still not improved in the non-parametric methods. This phenomenon was most prominent when the RS method was used. These results could be attributed to the limited sample size of reference individuals. In our study, the number of reference individuals in the 19-year-old age group of boys was less than 100 for both ALP and Cr, which is insufficient compared with the other age groups. In contrast, the WHO Multicenter Growth Reference Study enlarged the birth sample to 1737 to minimize the left edge effect [7]. It is particularly difficult to sample more reference individuals aged less than 1 year. Although we removed the reference individuals aged less than 1 year to lessen the edge effects, the sample size of infants is not sufficient. Therefore, a larger sample size would be needed to establish continuous RIs. In comparison with the non-parametric methods, the LMS and GAMLSS methods have fewer edge effects when sample size is relatively lacking. In addition, LMS and GAMLSS are easy to implement and have high accuracy, which could be factors to recommend them as convenient and accurate methods for clinical establishment of RIs.

Other factors besides age and sex, such as height and weight, may also affect analyte levels. In future research to establish RIs, multifactorial analysis could be considered. Further, the opinions of clinicians and laboratory physicians should be taken into consideration during the variable selection process. All of these directions would ultimately lead to huge challenges in terms of model selection and subject recruitment.

Moreover, different methods often estimated the upper and lower limits with the least amount of bias [1]. The idea of establishing reference limits with two different methods was previously explored by Horn PS et al. [28].

**Fig. 6:**
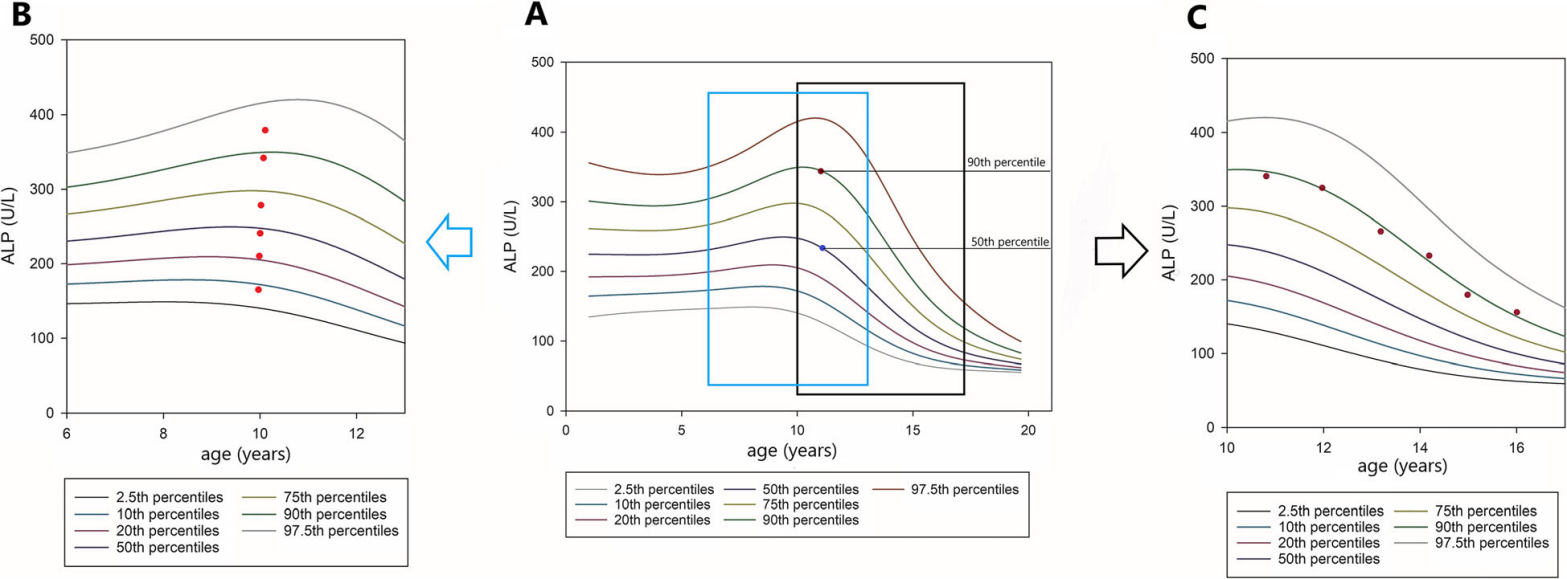
The application of continuous reference intervals. The seven curves denote the 97.5th, 90th, 75th, 50th, 20th, 10th and 2.5th percentiles for ALP of girls respectively. Dots show the result of individual laboratory examination. **a**. The percentile curves for ALP of boys aged 1 to 19 years. **b**. During hospitalization, patient’s ALP continued to increase for several times. **c**. With the increase of age, patient’s ALP decreased. ALP, alkaline phosphatase

There is a huge gap between the establishment of RIs and clinical practice. A possible solution is to integrate continuous RIs into laboratory testing platforms. The obtained models could be embedded into hospital clinical laboratory testing systems, and the RIs could be obtained from the models according to the information needed. Other quantile curves, such as the 5th, 25th, 50th, and 75th percentiles, can be easily obtained from the model. Therefore, doctors could not only judge whether the individual’s laboratory result is abnormal but also provide a graph to present the patient’s level compared with continuous RIs. In addition, longitudinal dynamic trends can be determined when individuals have multi-time laboratory results within a certain period (Fig. 6). Compared with a single test, the dynamic trends of some analytes could provide more diagnostic information about changes to individual health status. Moreover, graphical displays of clinical laboratory analytes would provide an improvement in clinical laboratory reporting.

The concept of continuous RIs is timeless and should become a standard throughout the entire field of laboratory medicine. It is necessary to establish continuous RIs for all ages rather than only focusing on the initial stages of life. When we are limited to the reference population, we cannot make such age divisions. Mørkrid et al. presented an elegant example of this viewpoint [29].

## Conclusions

Four statistical methods to estimate continuous RIs for ALP and Cr were simulated and verified. The verification of continuous RIs showed that all four methods could be used to establish continuous RIs of clinical laboratory analytes. The GAMLSS and LMS methods were more reliable than the RS and non-parametric methods, especially when sample size was insufficient. Therefore, the former two can be recommended as convenient and accurate methods for RIs establishment in clinical practice. In addition, the distribution of the data should be approximately normal when using the RS method to establish continuous RIs.

## Supplementary information

**Additional file 1.**

## Data Availability

The data are not publicly available as they contain information that could compromise research participant privacy. But they are available from the corresponding author on reasonable request. The request to access the raw data treated by deidentification from the PRINCE study must be approved by Academic Committee of PRINCE study.

## Abbreviations

ALP: Alkaline phosphatase
Cr: Serum creatinine
RIs: Reference intervals
OOR: Out of range
PRINCE: Pediatric Reference Interval in China study
CALIPER: Canadian Laboratory Initiative in Pediatric Reference Intervals
GAMLSS: The General Additive Model for Location Scale and Shape method
LMS: The Lambda-Median-Sigma method
RS: The radial smoothing method
BCT: The Box-Cox t distribution
BCPE: The Box-Cox power exponential distribution
