# Supplementary material for: Comparison of four algorithms on establishing continuous reference intervals for pediatric analytes with age-dependent trend
Source: BMC Med Res Methodol. 2020 Jun 1;20:136. doi: 10.1186/s12874-020-01021-y (PMC7268336; doi:10.1186/s12874-020-01021-y)
Supplement: Supplementary file 1 — Additional file 1. [file 12874_2020_1021_MOESM1_ESM.docx]

**Supplementary Material**


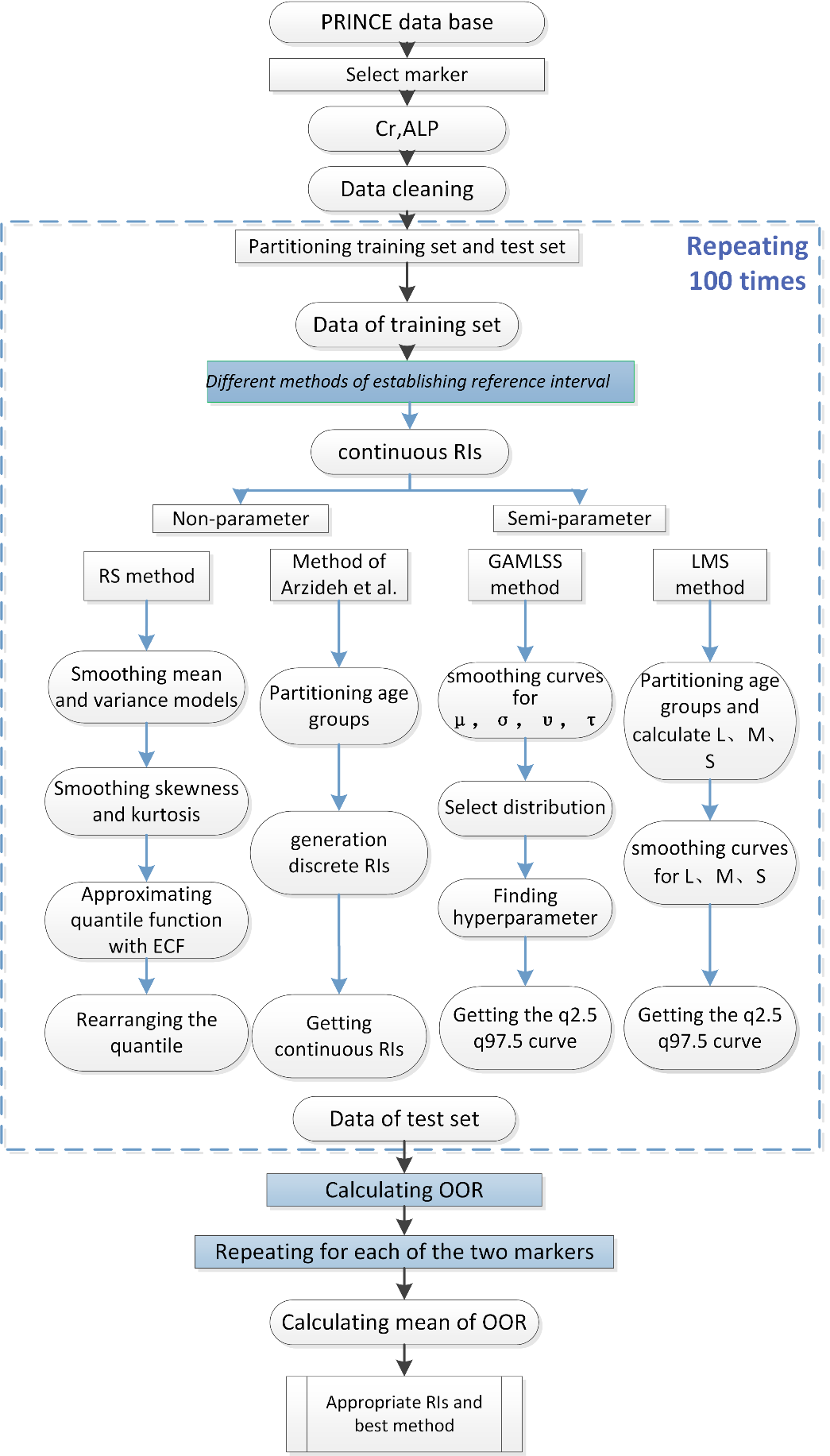


**Supplementary Fig. 1.** Statistical simulation process in this paper.


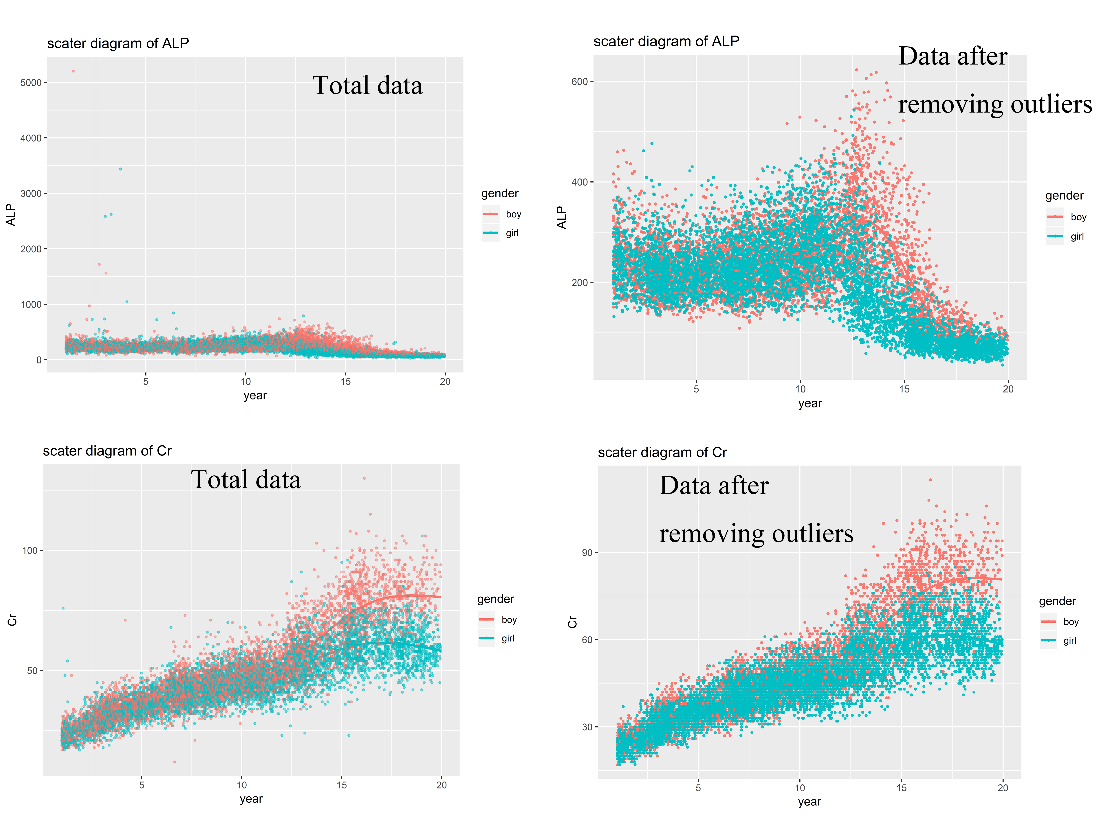


**Supplementary Fig. 2.** The [scatter](javascript:;) [diagram](javascript:;) of total data and data after removing outliers.


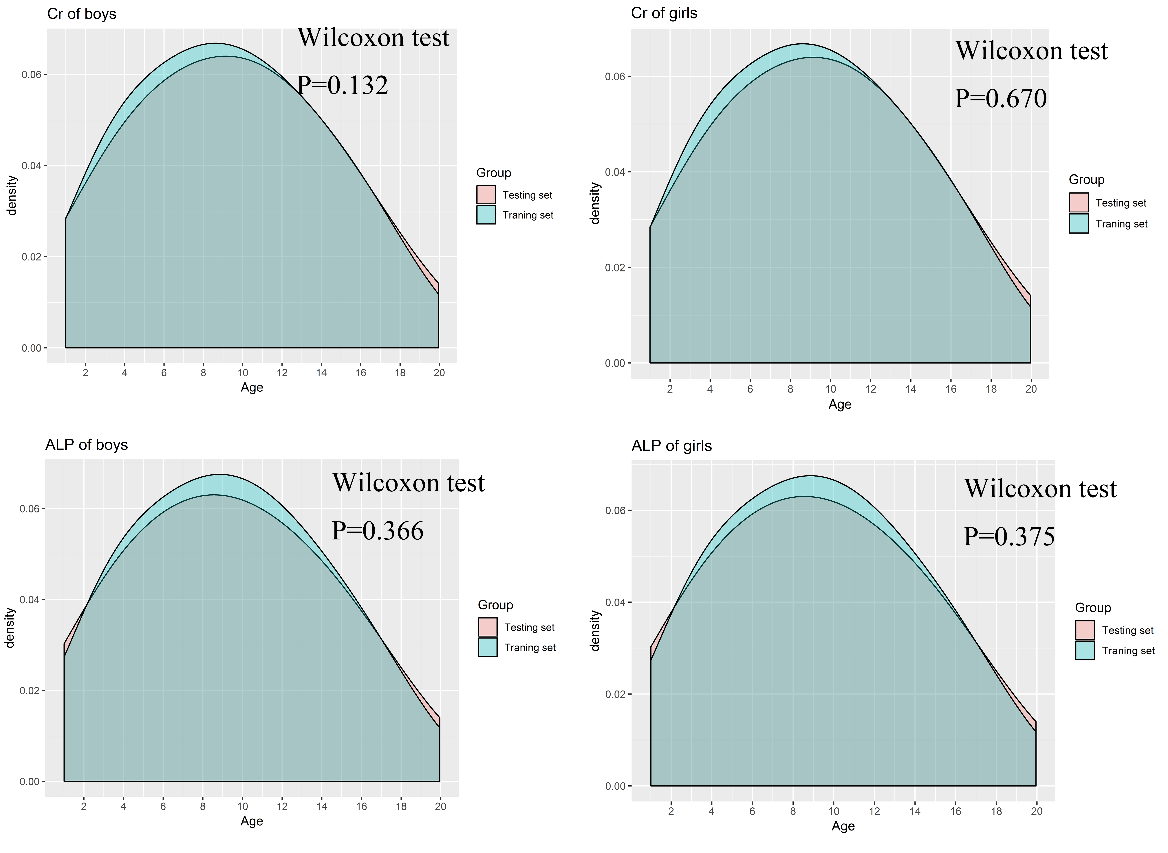


**Supplementary Fig. 3.** The age distribution of training and testing data sets*.*


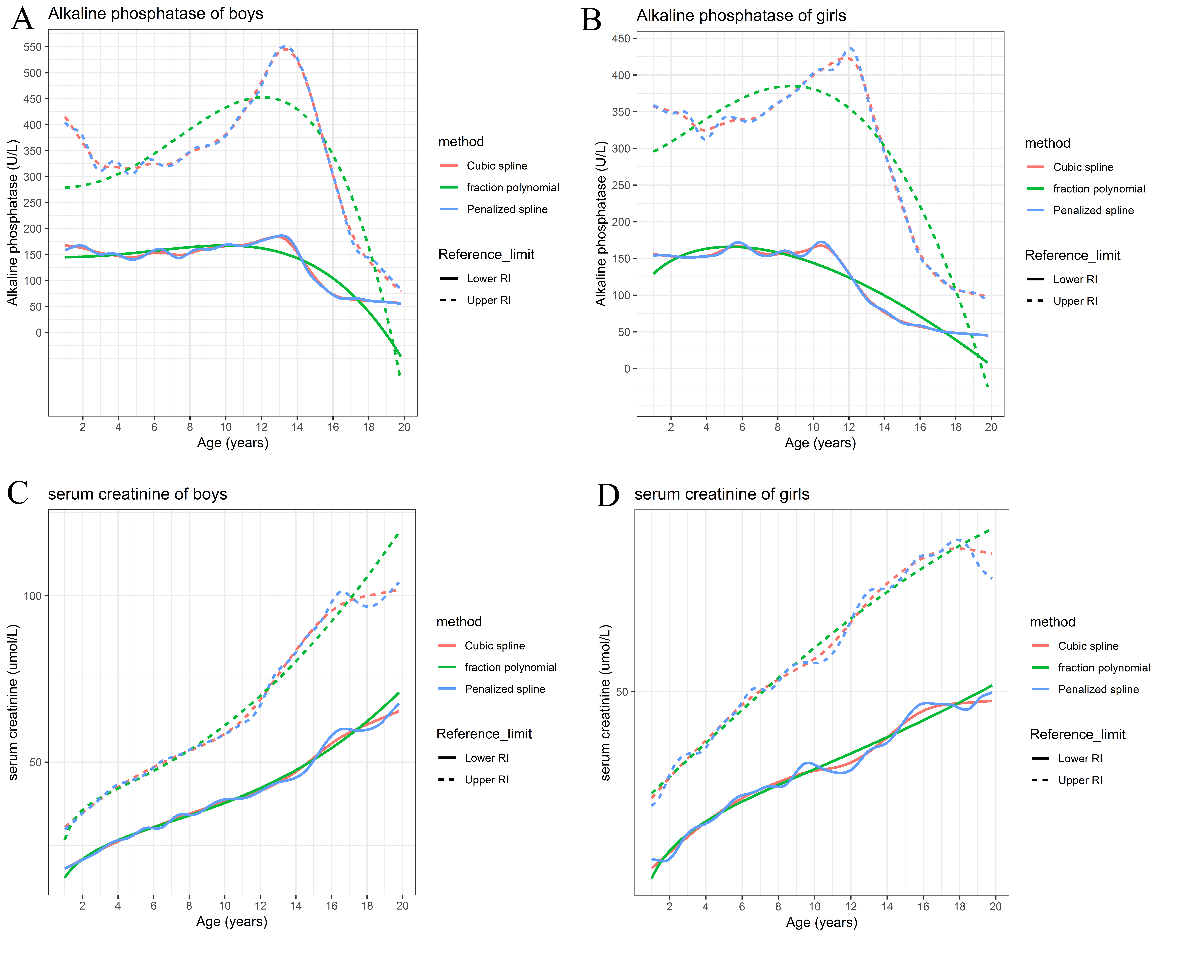


**Supplementary Fig. 4.** RIs of the improved non-parametric method with different smoothing method. Functions fitted by *gamlss* in R software without visual inspection.


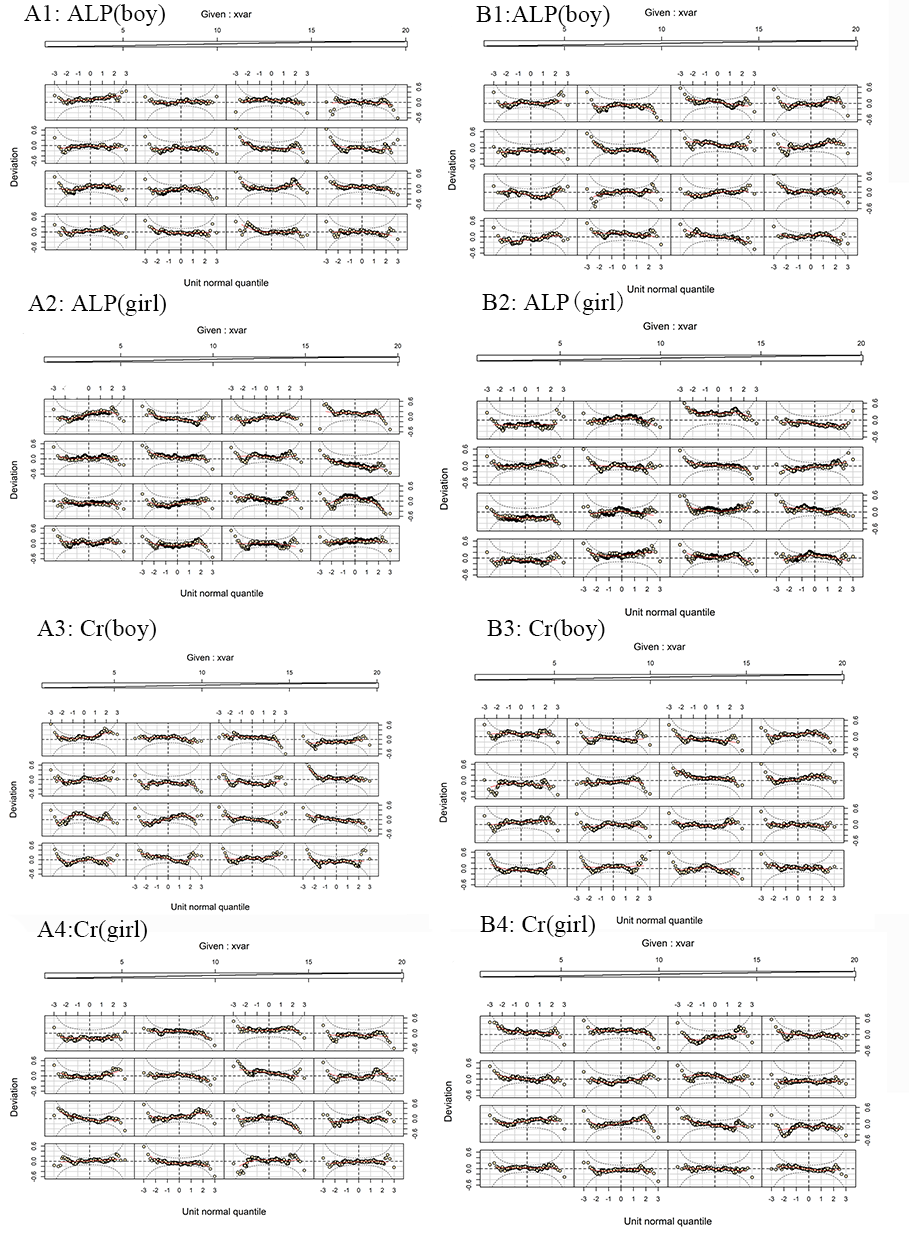


**Supplementary Fig. 5.** The worm plot of different simulation methods. (A1:A4) GAMLSS method (B1:B4) LMS method. Each plot consists of detrended Q-Q plots in 16 age groups.


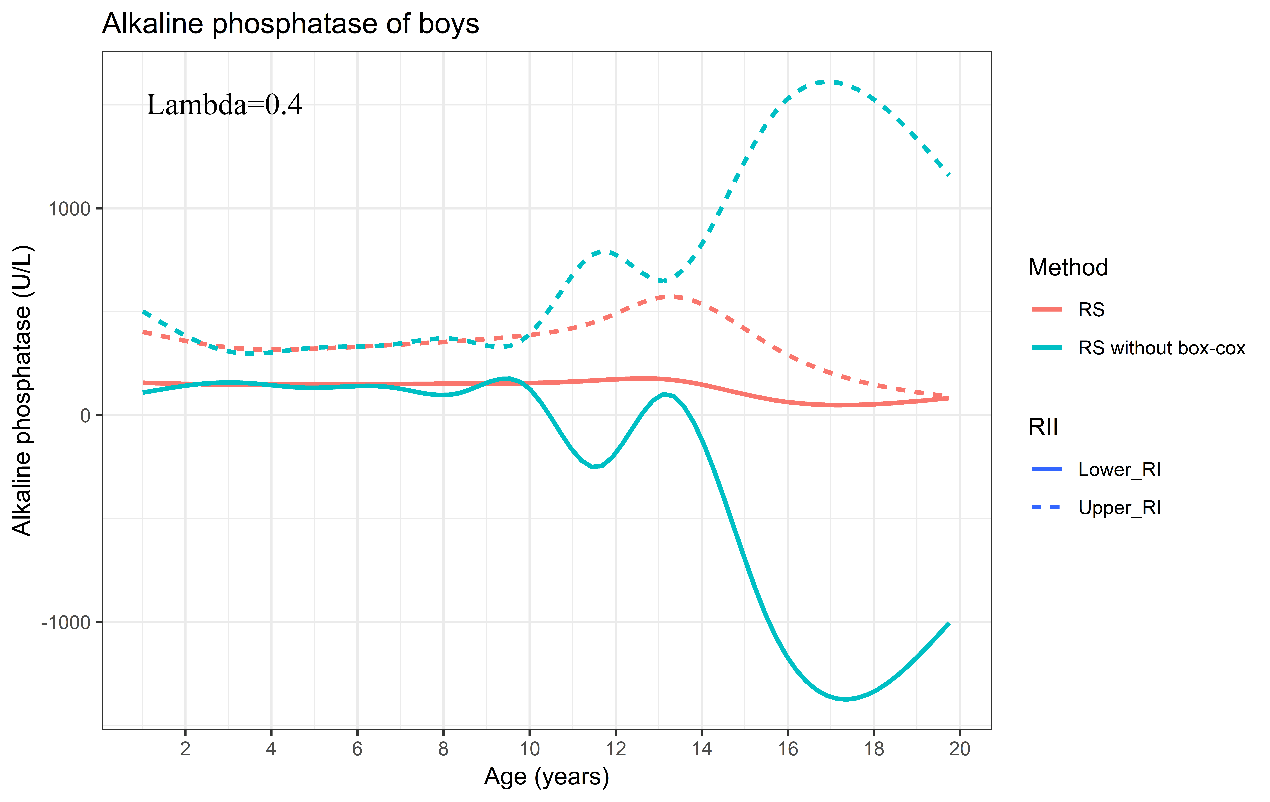


**Supplementary Fig. 6.** RIs of RS method with Box-Cox transformation. Lambda is the parameter of Box-Cox transformation estimated by maximum likelihood techniques.

**Supplementary Table 1.**

The Akaike information criterion of different smoothing methods in the improved non-parametric method.

| **^a^ Model** | **Cr (boy)** | | **Cr (girl)** | | **ALP (boy)** | | **ALP (girl)** | |
| --- | --- | --- | --- | --- | --- | --- | --- | --- |
|  | **mean** | **SD** | **mean** | **SD** | **mean** | **SD** | **mean** | **SD** |
| **Penalized splines** |  |  |  |  |  |  |  |  |
| Lower RI limit | 5122.00 | 211.05 | 5489.45 | 297.38 | 13581.67 | 157.03 | 13298.38 | 824.74 |
| Upper RI limit | 6461.38 | 935.21 | 6679.22 | 269.94 | 16891.16 | 275.96 | 18878.73 | 973.40 |
| **Cubic splines** |  |  |  |  |  |  |  |  |
| Lower RI limit | 7094.30 | 324.43 | 7963.03 | 205.84 | 17096.44 | 154.99 | 16930.54 | 262.29 |
| Upper RI limit | 9647.79 | 170.29 | 9153.13 | 207.82 | 21408.80 | 160.83 | 20837.66 | 230.70 |
| **Fraction polynomials** |  |  |  |  |  |  |  |  |
| Lower RI limit | 7704.72 | 337.91 | 8910.19 | 196.50 | 18641.18 | 135.00 | 19115.75 | 167.94 |
| Upper RI limit | 11357.64 | 162.22 | 10260.57 | 181.07 | 23864.07 | 126.91 | 23202.5 | 162.11 |

^a^ Smoothing parameters are selected by internal (i.e. local) maximum likelihood (ML) estimation.

**Supplementary Table 2.**

The AIC of different smoothing methods and distribution in GAMLSS method.

| **Model** | **Cr (boy)** | | **Cr (girl)** | | **ALP (boy)** | | **ALP (girl)** | |
| --- | --- | --- | --- | --- | --- | --- | --- | --- |
|  | **mean** | **SD** | **mean** | **SD** | **mean** | **SD** | **mean** | **SD** |
| **BCT distribution** |  |  |  |  |  |  |  |  |
| Cubic splines | 31471.20 | 67.76 | 29871.17 | 39.53 | 49481.08 | 349.80 | 50353.69 | 473.54 |
| Penalized splines | 29129.97 | 32.60 | 29701.95 | 34.41 | 48982.45 | 67.24 | 48416.59 | 37.01 |
| **BCPE distribution** |  |  |  |  |  |  |  |  |
| Cubic splines | 29252.79 | 32.43 | 29830.15 | 31.94 | 49450.63 | 80.37 | 49023.58 | 170.86 |
| Penalized splines | 29106.07 | 36.25 | 29680.95 | 23.76 | 48938.69 | 54.40 | 48380.56 | 43.12 |

^a^ AIC are calculated as mean of the result of 100 times simulation.

GAMLSS: General Additive Model for Location Scale and Shape; BCT: Box-Cox t; BCPE: Box-Cox power exponential.

**Supplementary Table 3.**

The age-specific OOR of GAMLSS method and continuous RIs for [alkaline](javascript:;) [phosphatase](javascript:;) .

| Age(years) | Boys | | | | Girls | | | | |
| --- | --- | --- | --- | --- | --- | --- | --- | --- | --- |
|  | Discrete RIs | | GAMLSS method | | Discrete RIs | | GAMLSS method | | |
|  | Lower OOR (%) | Upper OOR (%) | Lower OOR (%) | Upper OOR (%) | Lower OOR (%) | Upper OOR (%) | Lower OOR (%) | Upper OOR (%) |  |
| 1 | 0.00^a^ | 10.12 | 1.46 | 4.55 | 1.35 | 3.16 | 3.04 | 2.82 |  |
| 2 | 0.63 | 2.19 | 1.26 | 3.14 | 0.54 | 4.10 | 2.57 | 2.07 |  |
| 3 | 1.07 | 1.62 | 2.14 | 3.17 | 1.71 | 0.38 | 2.77 | 2.89 |  |
| 4 | 2.71 | 0.38 | 3.33 | 2.93 | 1.24 | 2.14 | 2.51 | 2.63 |  |
| 5 | 1.17 | 2.10 | 2.87 | 3.05 | 0.00 | 2.53 | 1.15 | 3.33 |  |
| 6 | 1.36 | 0.79 | 1.83 | 1.58 | 0.89 | 1.76 | 3.06 | 1.90 |  |
| 7 | 3.01 | 1.15 | 2.41 | 2.08 | 1.02 | 3.33 | 2.98 | 1.69 |  |
| 8 | 1.73 | 1.17 | 1.28 | 2.53 | 1.42 | 1.32 | 2.11 | 2.06 |  |
| 9 | 2.60 | 1.47 | 2.39 | 1.84 | 2.14 | 1.15 | 3.05 | 2.51 |  |
| 10 | 2.48 | 4.80 | 2.71 | 2.30 | 0.91 | 2.50 | 2.70 | 2.32 |  |
| 11 | 3.47 | 1.33 | 3.54 | 2.79 | 4.16 | 3.99 | 3.13 | 2.16 |  |
| 12 | 1.46 | 8.11 | 3.36 | 1.18 | 0.31 | 5.28 | 3.33 | 2.72 |  |
| 13 | 1.66 | 9.13 | 2.14 | 2.23 | 1.23 | 1.51 | 1.88 | 2.19 |  |
| 14 | 12.70 | 5.64 | 2.34 | 2.92 | 3.58 | 0.00 | 3.42 | 1.52 |  |
| 15 | 1.91 | 3.37 | 2.93 | 3.13 | 5.00 | 3.73 | 3.27 | 2.67 |  |
| 16 | 4.58 | 0.24 | 2.54 | 2.40 | 10.00 | 0.49 | 1.79 | 2.22 |  |
| 17 | 2.34 | 3.15 | 1.51 | 1.93 | 22.70 | 3.17 | 2.80 | 2.62 |  |
| 18 | 2.44 | 0.00 | 1.03 | 1.31 | 22.47 | 0.92 | 1.48 | 2.66 |  |
| 19 | 15.86 | 0.00 | 4.47 | 2.84 | 27.43 | 0.00 | 2.41 | 1.68 |  |

^a^ OOR are calculated as mean of the result of 100 times simulation.

GAMLSS, General Additive Model for Location Scale and Shape method; OOR, out of range.

**Supplementary Table 4.**

The age-specific OOR of GAMLSS method and continuous RIs for serum creatinine.

| Age  (years) | Boys | | | | Girls | | | |
| --- | --- | --- | --- | --- | --- | --- | --- | --- |
|  | Discrete RIs | | GAMLSS methed | | Discrete RIs | | GAMLSS methed | |
|  | Lower OOR (%) | Upper OOR (%) | Lower OOR (%) | Upper OOR (%) | Lower OOR (%) | Upper OOR (%) | Lower OOR (%) | Upper OOR (%) |
| 1 | 9.80^a^ | 0.00 | 4.13 | 2.86 | 9.55 | 0.06 | 5.53 | 3.44 |
| 2 | 1.20 | 4.29 | 3.96 | 2.27 | 0.00 | 3.79 | 6.57 | 5.16 |
| 3 | 3.36 | 0.90 | 2.61 | 2.87 | 0.46 | 0.00 | 3.32 | 1.45 |
| 4 | 0.00 | 2.88 | 1.85 | 2.50 | 0.60 | 5.50 | 5.58 | 3.37 |
| 5 | 0.00 | 6.32 | 1.42 | 2.10 | 3.35 | 0.55 | 3.79 | 3.16 |
| 6 | 4.05 | 0.42 | 4.16 | 2.10 | 0.95 | 2.78 | 4.39 | 4.54 |
| 7 | 0.08 | 1.38 | 1.93 | 2.56 | 0.63 | 4.55 | 5.72 | 2.97 |
| 8 | 0.75 | 3.58 | 4.12 | 2.78 | 4.31 | 1.66 | 6.51 | 4.38 |
| 9 | 1.73 | 0.50 | 1.11 | 2.44 | 0.24 | 2.94 | 2.87 | 3.76 |
| 10 | 1.02 | 2.73 | 2.17 | 3.11 | 0.62 | 1.48 | 4.10 | 1.07 |
| 11 | 0.52 | 4.33 | 1.87 | 1.66 | 1.97 | 5.56 | 6.16 | 2.28 |
| 12 | 3.15 | 0.33 | 2.32 | 3.32 | 2.60 | 0.93 | 2.11 | 3.26 |
| 13 | 1.49 | 1.53 | 2.88 | 2.52 | 1.72 | 3.23 | 2.89 | 3.11 |
| 14 | 1.35 | 9.19 | 2.78 | 1.63 | 1.34 | 4.42 | 1.85 | 1.38 |
| 15 | 3.73 | 2.06 | 2.40 | 1.80 | 1.77 | 2.19 | 1.86 | 2.22 |
| 16 | 1.54 | 5.51 | 2.77 | 2.18 | 1.72 | 3.06 | 1.65 | 1.61 |
| 17 | 1.22 | 3.48 | 1.68 | 2.87 | 1.20 | 4.88 | 1.61 | 2.43 |
| 18 | 0.52 | 2.07 | 3.09 | 2.22 | 2.27 | 3.37 | 4.47 | 2.38 |
| 19 | 0.00 | 10.19 | 2.00 | 2.77 | 0.15 | 0.92 | 2.80 | 1.28 |

^a^ OOR are calculated as mean of the result of 100 times simulation.

GAMLSS, General Additive Model for Location Scale and Shape method; OOR, out of range.
